# Enhancing viability and angiogenic efficacy of mesenchymal stem cells via HSP90*α* and HSP27 regulation based on ROS stimulation for wound healing

**DOI:** 10.1002/btm2.10560

**Published:** 2023-06-07

**Authors:** Inwoo Seo, Sung‐Won Kim, Jiyu Hyun, Yu‐Jin Kim, Hyun Su Park, Jeong‐Kee Yoon, Suk Ho Bhang

**Affiliations:** ^1^ School of Chemical Engineering, Sungkyunkwan University Suwon Republic of Korea; ^2^ Department of Systems Biotechnology Chung‐Ang University Anseong Republic of Korea

**Keywords:** angiogenesis, anti‐apoptosis, heat shock proteins, human mesenchymal stem cells, organic light‐emitting diode (OLED), wound healing

## Abstract

Light‐based therapy has been reported as a potential preconditioning strategy to induce intracellular reactive oxygen species (ROS) signaling and improve the angiogenic properties of various types of cells. However, bio‐stimulation mechanisms of light therapy in terms of ROS‐heat shock proteins (HSPs) mediated anti‐apoptotic and angiogenic pathways in human adult stem cells have not been fully delineated yet. Commonly used light sources such as light‐emitting diode (LED) and laser are accompanied by drawbacks, such as phototoxicity, thermal damage, and excessive ROS induction, so the role and clinical implications of light‐induced HSPs need to be investigated using a heat‐independent light source. Here, we introduced organic LED (OLED) at 610 nm wavelength as a new light source to prevent thermal effects from interfering with the expression of HSPs. Our results showed that light therapy using OLED significantly upregulated anti‐apoptotic and angiogenic factors in human bone marrow mesenchymal stem cells (hMSCs) at both gene and protein levels via the activation of HSP90α and HSP27, which were stimulated by ROS. In a mouse wound‐closing model, rapid recovery and improved re‐epithelization were observed in the light‐treated hMSCs transplant group. This study demonstrates that the upregulation of Akt (protein kinase B)‐nuclear factor kappa‐light‐chain‐enhancer of activated B cells (NF‐κB) signaling, caused by HSP90α and HSP27 expression, is the mechanism behind the anti‐apoptotic and angiogenic effects of OLED treatment on stem cells.

## 1 INTRODUCTION

In wound healing, angiogenesis is a key process necessary to supply oxygen and nutrients to wound sites.^1^, ^2^, ^3^ A number of cell therapies aiming to induce cytokines and growth factors required for angiogenesis to promote the repair of wound tissue have been reported.^4^, ^5^ Among various cell types, mesenchymal stem cell (MSC) is one of the most used cell types in cell therapy due to its immunomodulatory and high paracrine properties.^6^ However, the conventional cell injection method has a limitation in maintaining cell survival in local wound hypoxia, resulting in low therapeutic effects.^7^, ^8^ To achieve better angiogenic effects and higher viability of cells used in cell therapy, external materials, such as nanoparticles,^9^ hydrogels,^10^ growth factors,^11^ and cytokines^12^ have been introduced. However, they are still challenged by unwanted immune responses, degradation issues, and delivery methods, thus, limiting their use in clinical settings.^13^, ^14^, ^15^, ^16^

Given this background, there is an increasing demand for methods to improve the therapeutic efficacy of cells in a noninvasive and safe manner. Among various strategies, low‐level light therapy (LLLT) has been widely accepted as a useful preconditioning method to stimulate tissue regeneration.^17^, ^18^, ^19^, ^20^, ^21^ Previous reports have suggested that LLLT can induce angiogenic and extracellular matrix (ECM) secretion properties of cells by inducing ROS signaling, which is achieved by ROS generation following light absorption of cytochrome c oxidase in the mitochondrial respiratory chain.^22^, ^23^, ^24^, ^25^ However, light‐emitting diode (LED) and laser as generally used light sources^17^, ^18^, ^19^, ^20^, ^21^, ^22^, ^26^, ^27^, ^28^, ^29^ are accompanied by critical drawbacks such as nonuniform irradiation, thermal damage, and excessive ROS generation induction, which can be fatal to cell survival.^18^, ^26^, ^30^, ^31^ Also, there are conflicting results regarding the therapeutic efficacy of LLLT due to the lack of investigation about various photo‐parameters and corresponding cellular responses.^32^, ^33^, ^34^ Therefore, it is important to determine the appropriate mechanism associated with the light source used for LLLT.

Cells respond to a variety of external stresses by rapidly synthesizing heat shock proteins (HSPs).^35^, ^36^, ^37^ HSPs are a family of highly homologous chaperone proteins whose expression levels are increased after various types of stress, including ROS stimulation.^38^, ^39^, ^40^, ^41^ Representing 4%–6% of total proteins in stressed cells, HSPs participate in many essential cellular mechanisms by assisting transport, degradation, and folding of other proteins.^35^, ^36^, ^37^, ^42^, ^43^ HSP90α and HSP27 are the most studied HSPs. They are known to assist and stabilize cellular proteins related to anti‐apoptosis and angiogenesis.^40^, ^44^, ^45^, ^46^, ^47^, ^48^ Targeting HSPs during wound healing might have many important clinical implications for tissue repair. Several studies have reported that LED or laser irradiation with red/near‐infrared wavelength can increase expression levels of HSPs stimulated by ROS generation.^24^, ^49^, ^50^ However, bio‐stimulation mechanisms of LLLT in terms of ROS‐HSPs mediated anti‐apoptotic and angiogenic pathways in stem cells have not been fully delineated yet. In addition, since LED and laser are known to inevitably generate heat,^20^, ^26^, ^51^, ^52^ it is unclear whether increased expression levels of HSPs in previous studies are solely induced by ROS without any thermal effects.

HSPs can be sensitively affected by different types of stress other than ROS, such as heat stress.^35^, ^36^, ^37^ Therefore, when examining the mechanisms related to LLLT and HSPs, the light source needs to be carefully determined. Organic LED (OLED), which can irradiate light over large target areas more uniformly than LED, can be a good alternative as a low‐heat, high‐efficiency, and low‐power light source.^53^, ^54^, ^55^, ^56^ OLED has a broad spectrum similar to natural light. Thus, it might be better for inducing moderate ROS than LED and laser without causing phototoxicity.^53^, ^56^, ^57^ However, little is known so far about the therapeutic effect of LLLT using OLED on MSCs, which are ideal for controlling intracellular ROS production due to their high antioxidant capacity and ROS resistance.

**SCHEME 1:**
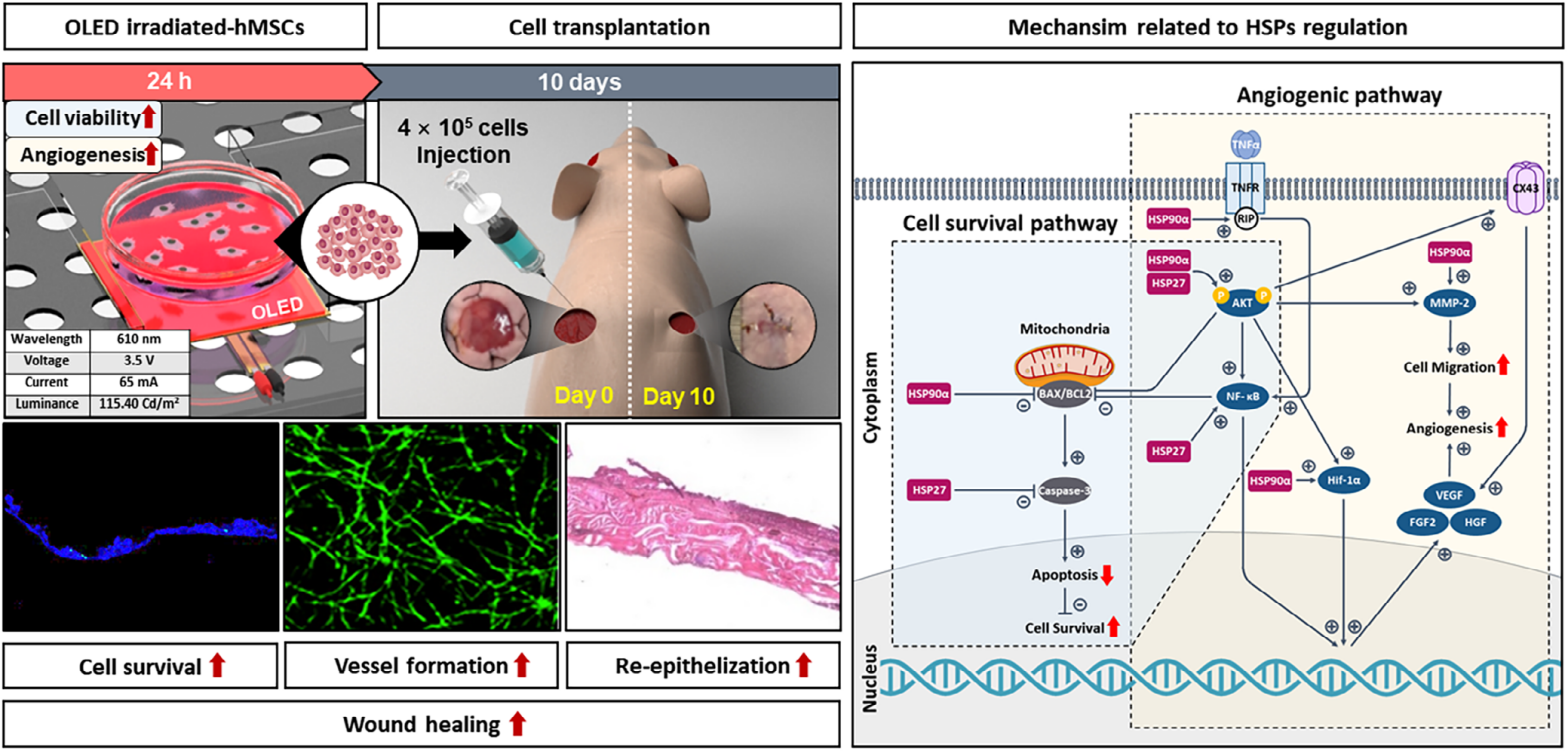
Schematic illustration of improved wound‐healing effect and underlying mechanism of organic light‐emitting diode (OLED)‐irradiated human bone marrow mesenchymal stem cells (hMSCs) therapy.

In this study, we investigate whether light therapy could enhance the wound‐healing effect of human bone marrow MSCs (hMSCs) in terms of expression of HSP90α and HSP27. To prevent thermal effects from interfering with the expression of HSPs, we introduced OLED that has not been applied to hMSCs before as an alternative light source with little heat generation. Another objective of this study was to suggest several mechanisms by which OLED therapy could affect the survival and angiogenesis of hMSCs in terms of HSP90α and HSP27. We found that HSP90α and HSP27 activated by ROS induced by OLED treatment modulate anti‐apoptosis and angiogenesis pathways by interacting with several key factors of each pathway. Furthermore, we confirmed angiogenic and cell survival improvement of light‐irradiated hMSCs, resulting in robust wound‐healing outcomes in vivo using a mouse wound‐closing model (Scheme 1).

## 2 RESULTS

### 2.1 Characteristics of OLED and optimization of irradiation time

**FIGURE 1:**
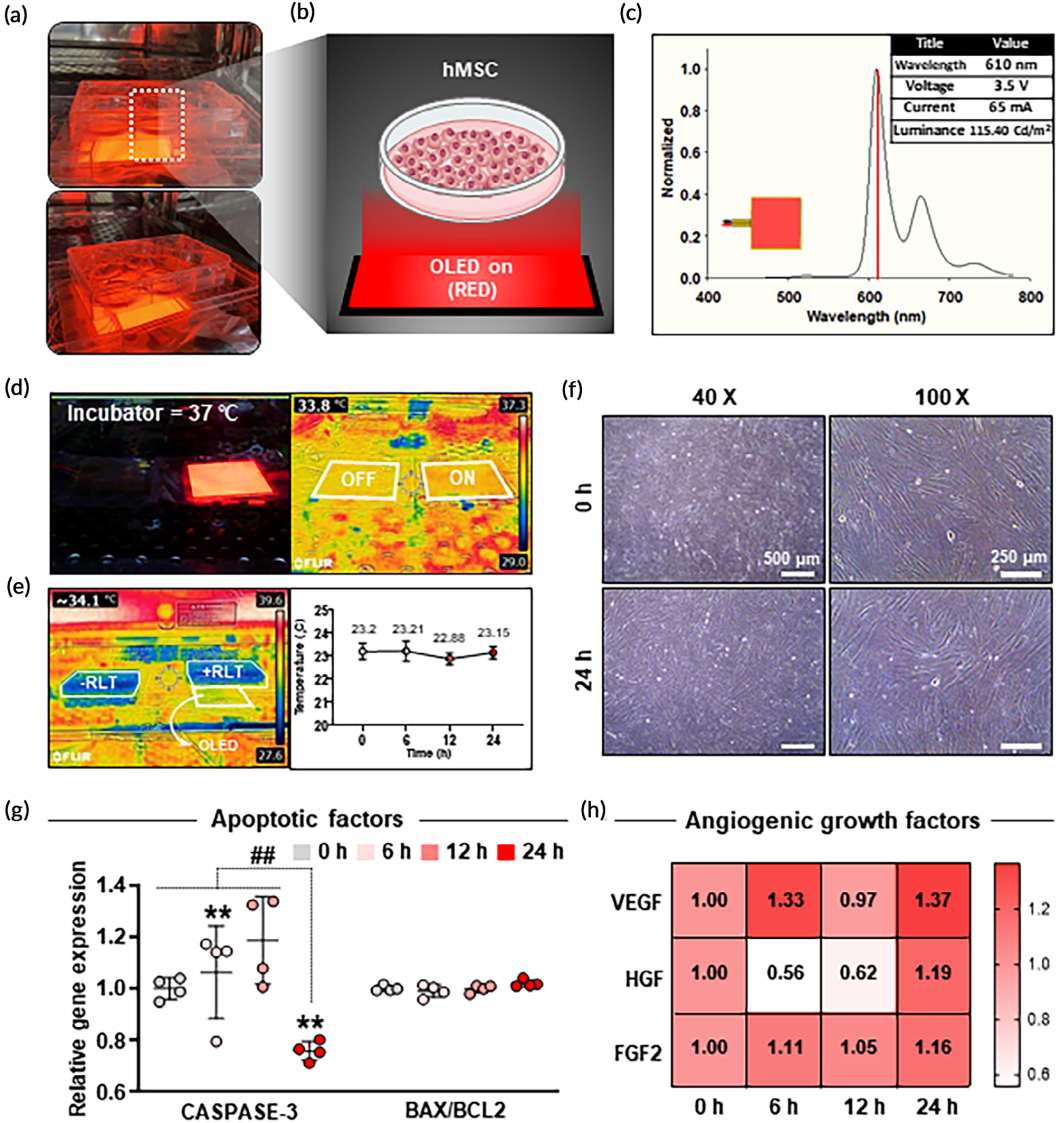
Characterization of organic light‐emitting diode (OLED) panel and optimization of irradiation time based on the expression of apoptosis‐ and angiogenesis‐related factors in human bone marrow mesenchymal stem cells (hMSCs). (a) Representative images and (b) illustration for light irradiation experimental setting. (c) Characteristics of OLED panel including peak wavelength, voltage, current, and luminance. (d) Thermal image of on/off‐OLED panel using a thermal imaging camera. (e) Temperature changes of culture medium after 0, 6, 12, and 24 h OLED irradiation using a real‐time infrared thermal imaging system. (f) Microscopic images showing the morphology of cells for 0 h (nonirradiated) and 24 h irradiated groups (scale bar = 500 μm for left image, 250 μm for right image). Relative gene expression levels of (g) apoptotic markers (CASPASE‐3, BAX/BCL2) and (h) angiogenic growth factors (vascular endothelial growth factor [VEGF], hepatocyte growth factor [HGF], and fibroblast growth factor 2 [FGF2]) after 0, 6, 12, and 24 h irradiation as evaluated by quantitative reverse transcription polymerase chain reaction (qRT‐PCR) (*n* = 3). Data are presented as mean ± SD. **p < 0.01 versus 0 h irradiated group and ##*p* < 0.01 versus 24 h irradiated group.

Experimental setup and characteristics of the OLED panel are shown in Figure 1a–c. We used 610 nm wavelength light (red) based on previous reports showing that LLLT with red/near‐infrared wavelength could positively affect proliferative and angiogenic efficacies of MSCs.^58^, ^59^ To determine if heat stress might cause any cell damage, a thermal imaging camera was used to capture heat image of the on/off‐OLED panel in an incubator (Figure 1d). No obvious change was observed in heat generation regardless of whether the panel was powered on or not. Temperature change of the culture medium was then tracked from 0 to 24 h of irradiation (Figure 1e). The temperature of the medium was kept almost constant for 24 h at 23°C. In addition, there was no difference in cell morphology between 0 and 24 h irradiated groups (Figure 1f). Thus, we can conclude that the OLED panel used in the experiment does not generate any significant heat that can cause cellular damage.

To find the optimal irradiation time, hMSCs were irradiated with light for 0, 6, 12, and 24 h. Expression levels of apoptosis‐ and angiogenesis‐related factors over time were measured for optimization. In the evaluation of apoptotic gene expressions, quantitative reverse transcription polymerase chain reaction (qRT‐PCR) analysis showed that the expression level of CASPASE‐3, a critical executioner of apoptosis,^60^ was increased over time from 0 to 12 h. However, it was significantly decreased at 24 h (Figure 1g). The ratio of BCL2‐associated X (BAX), an apoptosis regulator, to B‐cell lymphoma‐2 (BCL2) related to anti‐apoptosis is a marker of pro‐apoptosis.^61^ There was no statistically significant difference in the ratio of BAX to BCL2 between different time points. In the evaluation of expression levels of angiogenic genes, vascular endothelial growth factor (VEGF), hepatocyte growth factor (HGF), and fibroblast growth factor 2 (FGF2) showed the highest gene expression levels at 24 h after irradiation among different time points including the 0 h group (Figure 1h). Based on these results, we set 24 h as the optimized time, which was the most favorable time for cell viability and angiogenesis. Subsequent experiments were carried out by designating the 24 h red‐light treated group as the +RLT group and the nontreated group as the –RLT group.

### 2.2 Effect of RLT on HSP90α and HSP27 activation through ROS generation and apoptotic factor downregulation

**FIGURE 2:**
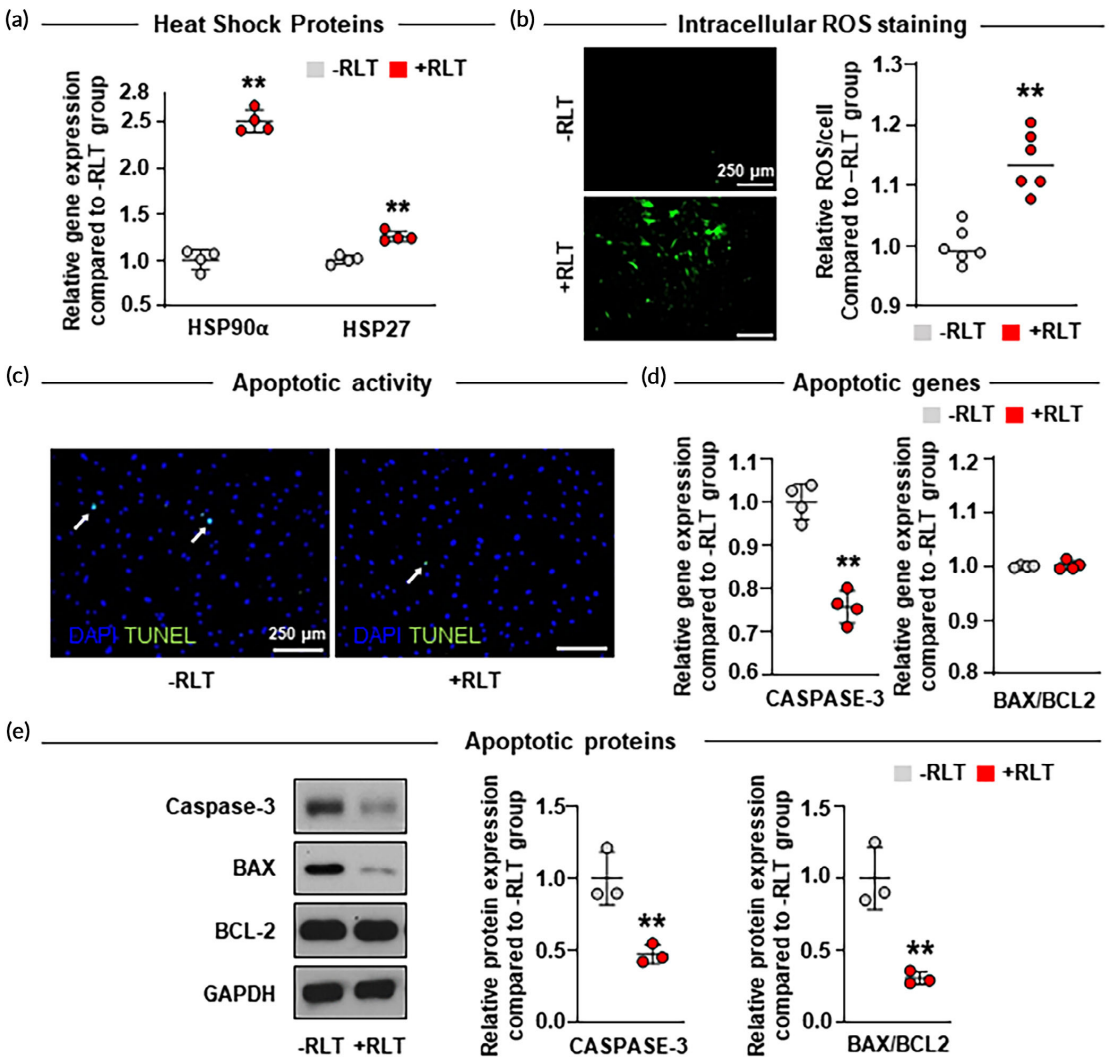
Effect of red light therapy (RLT) on HSP90α and HSP27 activation through ROS generation followed by apoptotic factor downregulation. (a) Relative gene expression levels of representative HSPs family (HSP90α, HSP27) in human bone marrow mesenchymal stem cells (hMSCs) (−RLT) and RLT‐treated hMSCs (+RLT) as evaluated by quantitative reverse transcription polymerase chain reaction (qRT‐PCR) analysis (*n* = 4). (b) Intracellular ROS staining using DCF‐DA (green) (scale bar = 250 μm) and its quantification expressed as relative (percent) fluorescence intensities. (c) Apoptotic activities in –RLT and +RLT groups as evaluated by TUNEL assay (apoptotic cells [white arrows] in green [scale bar = 250 μm]). Relative (d) gene and (e) protein expression levels of apoptotic factors (CASPASE‐3, BAX/BCL2) in –RLT and +RLT groups as evaluated by qRT‐PCR (*n* = 4) and western blotting analysis (*n* = 3), respectively. The uncropped full‐length blots are presented in Figure S1. Data are presented as mean ± SD. **p < 0.01 versus –RLT group.

The molecular pathway of light‐based therapy has been mainly reported as ROS signaling by means of ROS generation.^22^, ^23^, ^24^, ^25^ The production of ROS can lead to an activation of HSPs known to protect cells from damage and facilitate recovery.^38^, ^39^, ^40^, ^41^ To investigate whether RLT‐affected HSPs activity, we analyzed expression levels of HSP90α and HSP27 as representative HSPs family in hMSCs with and without RLT (Figure 2a). qRT‐PCR analysis showed that HSP90α and HSP27 expression levels in the +RLT group were increased by 2.5 and 1.25 times, respectively, compared to those in the –RLT group. To determine whether increases of HSP90α and HSP27 expression levels were due to ROS generation, intracellular ROS staining was performed (Figure 2b). Results revealed that a higher intracellular level of ROS was detected in the +RLT group than in the –RLT group. Since we previously confirmed that OLED had little effect on heat during thermal evaluation (Figure 1d,e), we could conclude that the upregulation of HSP90α and HSP27 was attributed to ROS generation rather than heat.

Induction of HSPs is known to protect cells from a diverse array of stresses. The cytoprotective function of HSP90α and HSP27 is largely due to their anti‐apoptotic function by interacting with different key apoptotic proteins. It has been shown that HSP90α is associated with the anti‐apoptotic protein BCL2, resulting in BAX inactivation.^45^, ^46^ HSP27 can also essentially block caspase‐dependent apoptotic pathways by inhibiting the formation of CASPASE‐3 activation complex.^45^, ^46^, ^62^, ^63^, ^64^ Therefore, we next examined whether the upregulation of HSP90α and HSP27 in RLT‐treated hMSCs was involved in the regulation of factors within the apoptotic cascade. To investigate changes in viability of hMSCs by RLT, apoptotic activity in hMSCs with or without RLT was detected by TUNEL staining (Figure 2c). Results revealed a lower amount of apoptotic cells in the +RLT group than in the –RLT group. Apoptotic gene and protein expression levels were then determined by qRT‐PCR and western blotting analysis, respectively. Quantification was then performed to compare cytotoxicity between the two groups (Figure 2d,e). Significant decreases of both gene and protein expressions of CASPASE‐3 were observed in the +RLT group compared to those in the –RLT group. In particular, protein expression of CASPASE‐3 in the +RLT group was twice smaller than that in the –RLT group. The expression of BAX/BCL2 showed no appreciable change between the two groups at the gene level. However, at the protein level, BAX/BCL2 expression decreased significantly to less than half of that in the +RLT group. Overall, we concluded that induction of HSP90α and HSP27 triggered by light stimuli could enhance the survival of hMSCs by inhibiting apoptotic factors within the apoptosis pathway.

### 2.3 Key client factors of HSP90α and HSP27 within apoptotic and angiogenic cascades

HSPs are multi‐domain chaperone proteins that can play significant roles in cell physiology as intra‐ and extracellular signaling ligands. In addition to their survival‐promoting effects as observed above, the activity of HSPs can modulate multiple events within angiogenic pathways. Key elements within the angiogenic cascade, such as Akt (protein kinase B) and nuclear factor kappa‐light‐chain‐enhancer of activated B cells (NF‐κB), are also known to be subject to modulation by HSP90α and HSP27.^45^, ^46^, ^47^, ^48^, ^65^, ^66^, ^67^, ^68^

**FIGURE 3:**
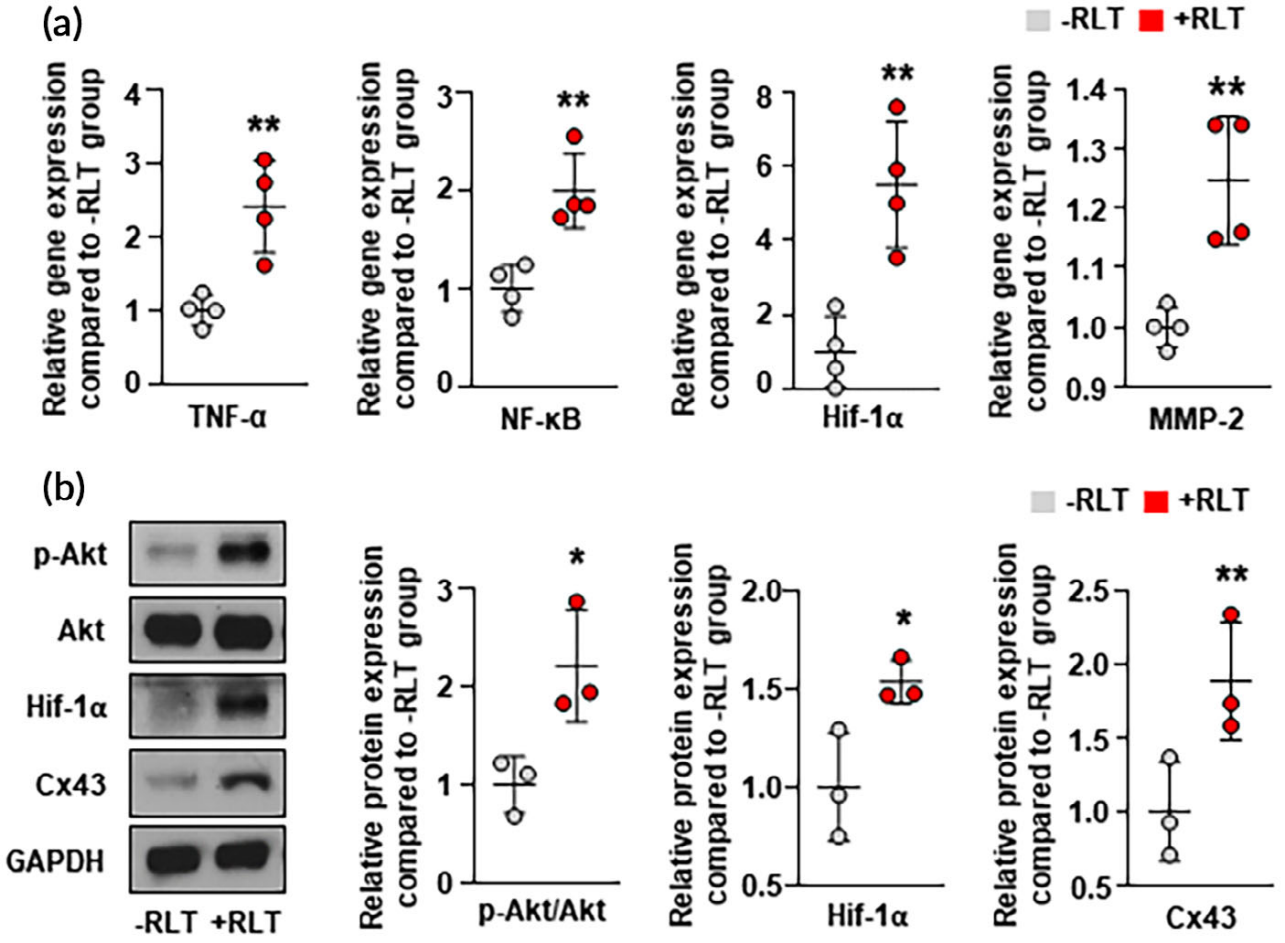
Key client factors of HSP90α and HSP27 within apoptotic and angiogenic cascades. (a) Relative gene expression levels of tumor necrosis factor‐alpha (TNF‐α), nuclear factor kappa‐light‐chain‐enhancer of activated B cells (NF‐κB), hypoxia‐inducible factor 1‐alpha (HIF‐1α), and matrix metalloproteinase‐2 (MMP‐2) in –RLT and +RLT groups as evaluated by quantitative reverse transcription polymerase chain reaction (qRT‐PCR) analysis (*n* = 4). (b) Relative protein expression levels of phosphorylated Akt (p‐Akt), Akt, HIF‐1α, and CX43 in –RLT and +RLT groups as evaluated by western blotting analysis (*n* = 3). The uncropped full‐length blots are presented in Figure S1. Data are presented as mean ± SD. *p < 0.05, **p < 0.01 versus –RLT group.

To delineate molecular mechanisms of RLT in association with HSPs, we examined client proteins of HSP90α and HSP27 operating in angiogenic signal transduction pathways through qRT‐PCR and western blotting analysis (Figure 3a,b). The expression of tumor necrosis factor‐alpha (TNF‐α) known to activate NF‐κB by binding to its receptor^69^, ^70^, ^71^ showed a significant increase in the +RLT group than in the –RLT group. NF‐κB expression, which could promote cell survival and angiogenesis,^72^, ^73^, ^74^, ^75^ was shown to be almost two‐fold higher in the +RLT group than that in the –RLT group. Expression levels of hypoxia‐inducible factor 1‐alpha (HIF‐1α)^76^, ^77^, ^78^, ^79^ and matrix metalloproteinase‐2 (MMP‐2)^80^, ^81^, ^82^ as important regulators of angiogenesis and cell migration were increased 5 and 1.25 times in the +RLT group, respectively, compared to those in the –RLT group. In western blotting analysis, phosphorylated Akt (p‐Akt) expression known to play a pivotal role in modulating cellular signaling for cell survival and angiogenesis,^83^, ^84^, ^85^, ^86^ was more than 2‐fold higher in the +RLT group than that in the –RLT group. Expression levels of HIF‐1α and connexin43 (Cx43)^87^, ^88^ associated with angiogenesis and cell migration were increased 1.5 and 2 times in the +RLT group, respectively, compared to those in the –RLT group. These results suggest that activation of HSP90α and HSP27 triggered by RLT can promote angiogenesis and cell survival by activating their client proteins.

### 2.4 Enhanced angiogenic capacity of RLT‐treated hMSCs

**FIGURE 4:**
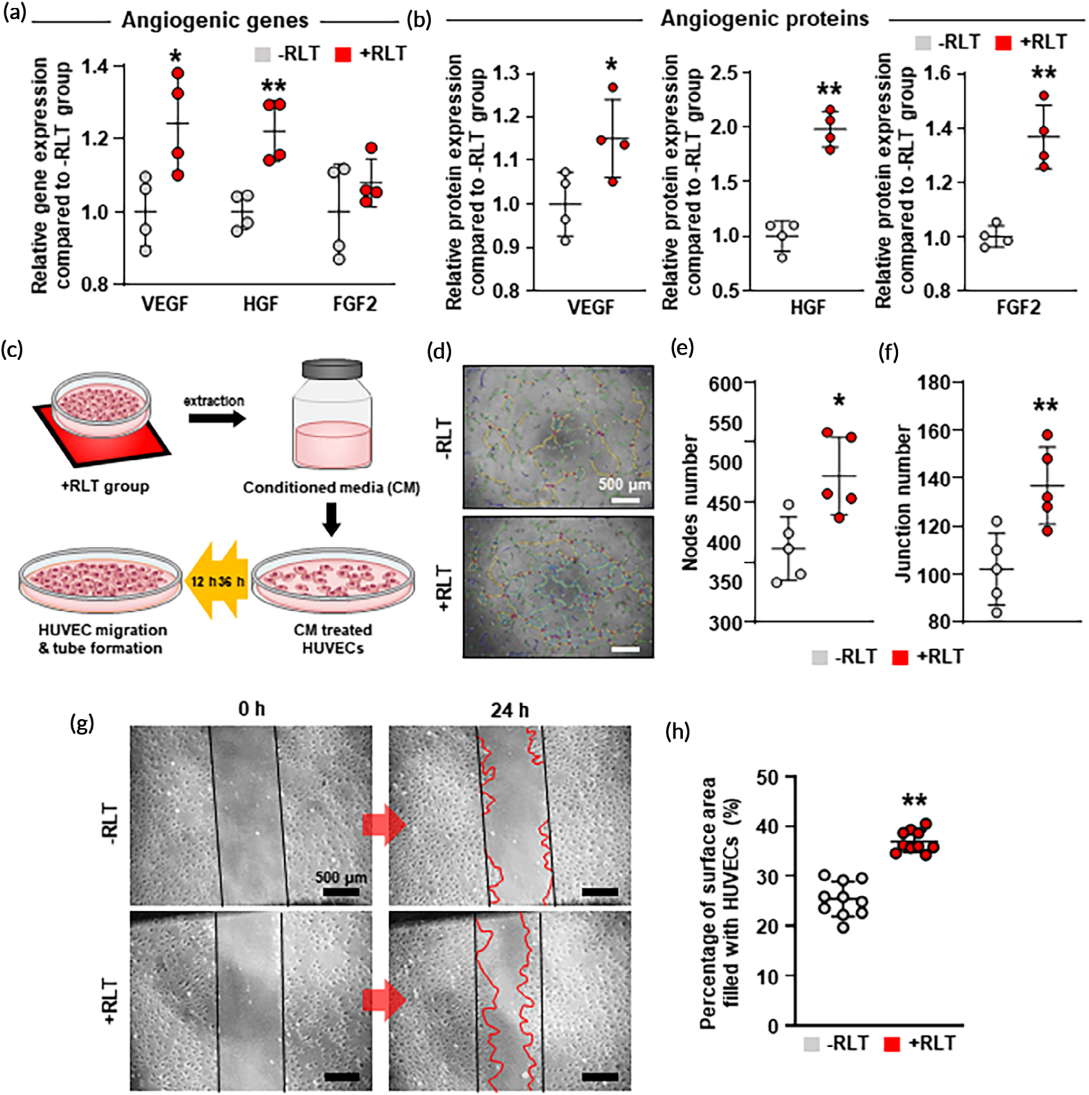
Enhanced angiogenic capacity of RLT‐treated human bone marrow mesenchymal stem cells (hMSCs) as evaluated by upregulation of angiogenic paracrine factor secretion. Relative (a) gene and (b) protein secretion of angiogenic growth factors (vascular endothelial growth factor [VEGF], hepatocyte growth factor [HGF], and fibroblast growth factor 2 [FGF2]) in –RLT and +RLT groups as evaluated by quantitative reverse transcription polymerase chain reaction (qRT‐PCR) (*n* = 4) and enzyme‐linked immunosorbent assay (ELISA) analysis (*n* = 4). (c) Schematic diagram of conditioned medium (CM) collection and treatment of human umbilical vein endothelial cells (HUVECs) with CM to evaluate tube formation and migration. (d) Image of tube formation by HUVECs after CM treatment (scale bars = 500 μm). (e–f) Quantification of the number of junctions (*n* = 4) and nodes (*n* = 4) in each group. (g) Image of migrated HUVECs treated with CM from –RLT and +RLT groups using scratch assay, and (h) Quantification by measuring filled area in each group (*n* = 12, scale bars = 500 μm). Data are presented as mean ± SD. *p < 0.05, **p < 0.01 versus –RLT group.

We next determined whether RLT could enhance the expression of angiogenic paracrine factors in hMSCs. Expression levels of VEGF, HGF, and FGF2 as representative angiogenic markers were quantified by qRT‐PCR and ELISA (Figure 4a,b). Results indicated that gene expression and protein secretion of VEGF, HGF, and FGF2 were all upregulated in the +RLT group compared to those in the –RLT group. We further quantified the angiogenic efficacy of hMSCs after RLT to assess whether secreted angiogenic growth factors in each group could stimulate tube formation and migration of endothelial cells. Human umbilical vein endothelial cells (HUVECs) were treated with conditioned medium (CM) extracted from –RLT or +RLT group (Figure 4c). As observed in Figure 4d, HUVECs treated with CM from the +RLT group showed a denser tubular network structure by forming more nodes (Figure 4e) and junctions (Figure 4f) than those treated with CM from the –RLT group. HUVEC migration efficacy was also evaluated by wound scratch assay (Figure 4g). As observed in Figure 4h, the percentage of the surface area filled with HUVECs treated with CM from the +RLT group reached almost 40%, whereas that filled with HUVECs treated with CM from the –RLT group reached less than 30%. These results suggest that angiogenesis and migration efficacy were enhanced by angiogenic paracrine factors secreted by RLT‐treated hMSCs.

### 2.5 In vivo wound‐healing efficacy of transplanted RLT‐treated hMSCs

**FIGURE 5:**
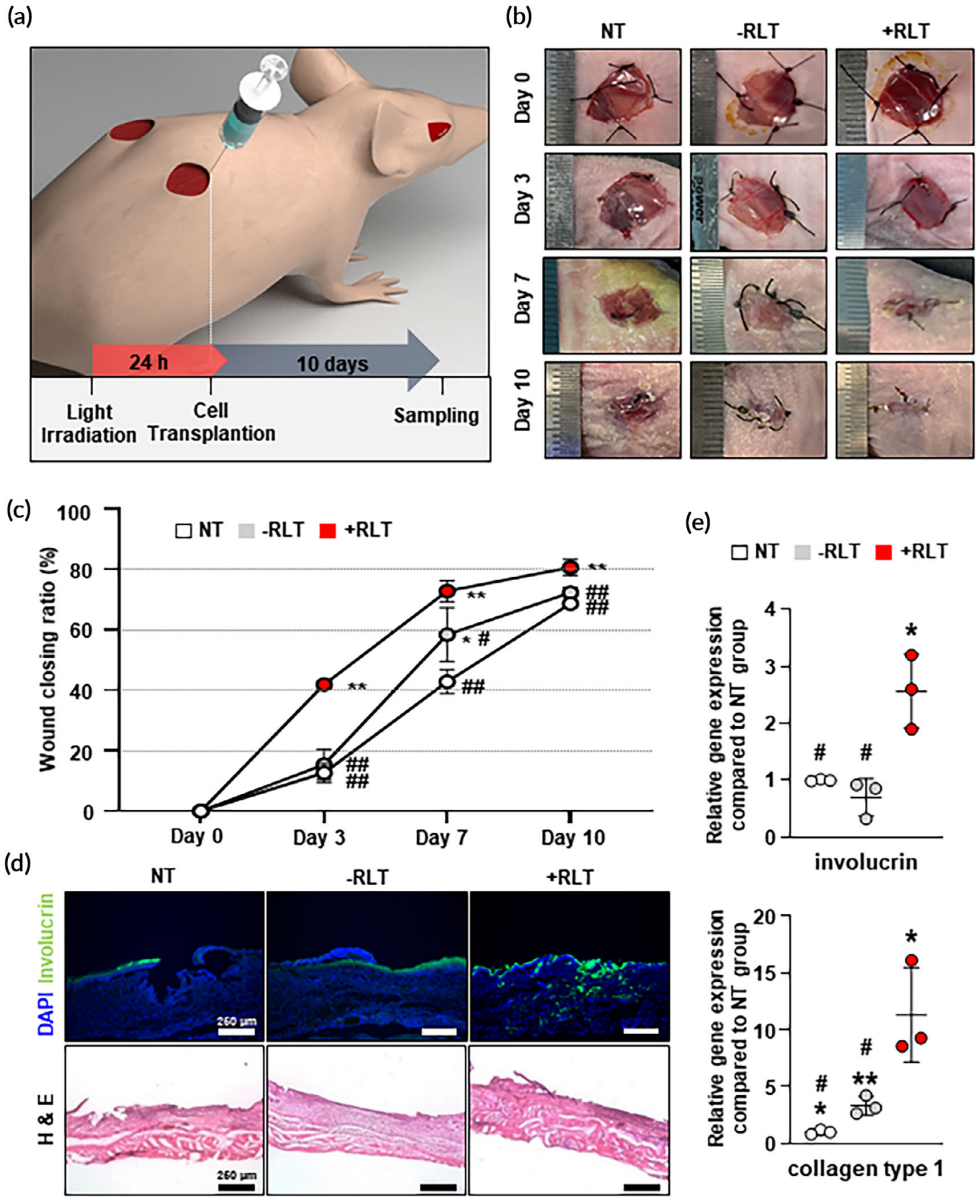
In vivo wound‐healing efficacy of transplanted red light therapy (RLT)‐treated human bone marrow mesenchymal stem cells (hMSCs) in a mouse wound‐closing model. (a) Schematic illustration of the procedure used to treat a mouse skin wound‐healing model with RLT‐treated hMSCs. (b) Representative wound‐closing physiology and (c) wound‐closing ratios of no‐treatment (NT), hMSCs injection (−RLT), and RLT‐treated hMSCs injection (+RLT) groups on days 0, 3, 7, and 10 after each transplantation (*n* = 6). (d) Double immunofluorescent staining with DAPI and involucrin in skin tissues (scale bar = 250 μm) and H&E staining of wound tissue sections from each group at 10 days after the treatment (scale bars = 250 μm). (e) Relative gene expression levels of involucrin and collagen type1 from mouse wound region as evaluated by quantitative reverse transcription polymerase chain reaction (qRT‐PCR) compared to those in the NT group (*n* = 3). Data are presented as mean ± SD. *p < 0.05 and **p < 0.01 versus NT group; #*p* < 0.05 and ##*p* < 0.01 versus +RLT group.

We applied RLT‐treated hMSCs to a mouse wound model to find out whether they had better wound‐healing efficacy than conventional cell therapies. Figure 5a summarizes the procedure for the whole in vivo test. After cell transplantation, wound closure rates were observed at multiple time points (0, 3, 7, and 10 days) (Figure 5b, c). In contrast to the NT group recovering at a constant rate, the –RLT group and the +RLT group showed rapid wound recovery rates between 3–7 days and between 0–7 days, respectively. At 10 days after treatment, the wound in the +RLT group had recovered more than 80% (80.7%), which was higher than that in the –RLT group (72.4%) or the NT group (68.7%).

The wound‐healing efficacy was further assessed by immunohistochemistry and histology (Figure 5d). Involucrin, a marker expressed in epidermal keratinocytes, is known to form a cornified envelope by crosslinking with other structural proteins.^89^, ^90^, ^91^ Among different groups, the +RLT group showed the highest involucrin signals, indicating an enhancement in re‐epithelization. In hematoxylin and eosin (H&E) staining, which provided histological observation of healed tissues, the +RLT group exhibited significant improvement related to skin epithelization in the epidermis and basal layer. These results were supported by qRT‐PCR analysis of wound tissues (Figure 5e). Collagen type 1, a key component of the extracellular matrix, is important during proliferative and remodeling phases of wound healing.^92^, ^93^, ^94^ Significant upregulation of involucrin and collagen type 1 was identified in the +RLT group compared to NT and –RLT groups. In conclusion, RLT could promote the therapeutic efficacy of hMSCs better than conventional cell therapy by not only accelerating wound closure, but also effectively maintaining the skin layer itself.

### 2.6 Enhanced cell survival and angiogenesis in RLT‐induced ROS stimulation via regulating HSP90α and HSP27 pathways

Overall, we showed that hMSCs therapy using RLT could enhance tissue regeneration from the perspective of cell viability and angiogenesis. This regulation was confirmed to have partially occurred through the activation of HSP90α and HSP27 induced by ROS. HSP90α and HSP27 have attracted attention due to their cytoprotective and angiogenesis‐inducing properties.^40^, ^44^, ^45^, ^46^, ^47^, ^48^ The regulatory role of HSP90α and HSP27 depends on their ability to correlate with multiple intracellular clientele.^35^, ^36^, ^37^, ^42^, ^43^ Their client proteins include a wide range of proteins, such as signal transducing kinase and ligand‐dependent transcription factors including Akt and NF‐κB known to play a pivotal role in anti‐apoptotic and angiogenic processes.

**FIGURE 6:**
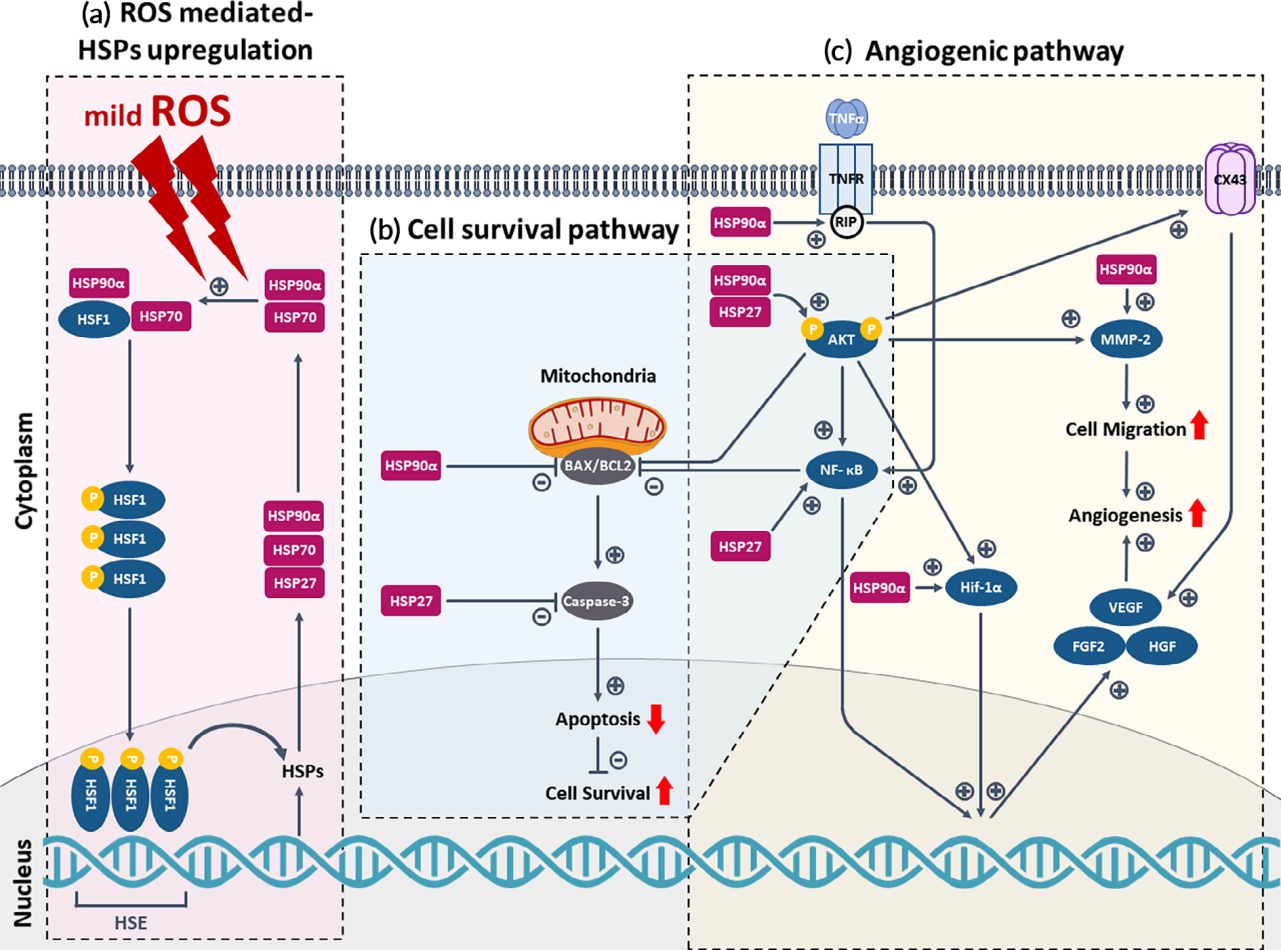
Enhanced cell survival and angiogenesis in red light therapy (RLT) induced‐reactive oxygen species (ROS) stimulation via regulating HSP90α and HSP27 pathways. Two‐way cell signaling pathway via (a) ROS‐mediated‐heat shock proteins (HSPs) upregulation: (b) Cell survival and (c) angiogenesis.

We here report the mechanism of RLT in respect of HSPs based on experimentally identified factors (Figure 6). In response to ROS stimulation by light, HSPs are known to be rapidly synthesized and accumulated in cells by heat shock factors 1 (HSF1).^38^, ^39^, ^44^, ^95^ HSP90α and HSP27 expression can be strongly induced in this process. The sequence of events mediated by HSP90α and HSP27 in RLT‐treated hMSCs can be broadly categorized into two pathways: cell survival pathway and angiogenic pathway. Both pathways are mainly driven by the regulation of Akt‐NF‐κB signaling. The binding of HSP90α is known to protect Akt from dephosphorylation, resulting in phosphorylated form of Akt (p‐Akt).^65^, ^66^ Activation of p‐Akt signaling can lead to anti‐apoptosis by decreasing the level of BAX/BCL2^45^, ^46^ as well as angiogenic cascade by increasing expression levels of HIF‐1α, MMP‐2, and Cx43.^5^, ^83^, ^84^, ^85^, ^86^, ^96^, ^97^ p‐Akt is also known to participate in NF‐κB activation by phosphorylating IkB kinase, resulting in promotion of NF‐κB‐mediated anti‐apoptosis and angiogenesis.^98^ Another route by which HSP90α can affect NF‐κB activation is via receptor‐interacting protein (RIP) stabilization. HSP90α is known to directly interact with RIP, an essential component in TNF‐α‐induced activation, which permits TNF‐α‐induced NF‐κB activation.^99^, ^100^ Although TNF‐α can induce cell death, its death‐inducing ability plays a minor role compared to other apoptotic proteins. HSP90α can also directly bind and activate MMP2 via the hemopexin domain, which facilitates cell migration and angiogenesis.^96^ HIF‐1α, a critical transcription factor for inducing angiogenic paracrine factors and migration, is activated by p‐Akt^5^ and HSP90α.^101^ The gap junction protein Cx43 is known to be activated by p‐Akt signaling during wound repair to induce the migration of keratinocytes to the wound site by gap junction communication.^97^ Cx43 can also mediate the upregulation of MMP2 and VEGF for angiogenesis under the control of HSP90α.^87^, ^88^ HSP27 is also known to contribute to anti‐apoptosis by inhibiting the formation of the CASPASE‐3 activation complex, leading to its inactivation and cell survival.^45^, ^46^, ^62^, ^63^, ^64^ HSP27 can bind to Akt and form a signaling complex necessary for Akt activation in stressed cells.^67^ HSP27 can directly mediate the enhancement of NF‐κB activity by promoting the degradation of IkB, which results in NF‐κB cell survival and angiogenesis signals.^68^ Moreover, HSP27 can promote angiogenesis and cell migration in blood via upregulating VEGF gene transcription.^102^ In summary, HSP90α and HSP27 activated by RLT might intervene at multiple points in both anti‐apoptotic and angiogenic pathways, thereby enhancing the wound‐healing efficacy of hMSCs.

## 3 DISCUSSION

Clinical obstacles faced by conventional cell therapy include low viability of transplanted cells due to harsh conditions of wound tissue, which can reduce cell engraftment.^7^, ^8^ In addition, since defected wounds lack oxygen and nutrients, enhanced angiogenic paracrine effect with maintaining cell viability is an important determinant of healing outcomes.^1^ Growing evidence suggests that LLLT might cause angiogenic properties of cells by inducing oxidative stress.^17^, ^18^, ^19^, ^20^, ^21^ Although it has been clinically applied for various purposes, investigation between undeterminate photo‐variables and corresponding intracellular responses is relatively insufficient. Also, there are several contrasting reports showing that commonly available LED and laser do not always guarantee a beneficial effect due to nonuniform irradiation and excessive ROS induction. They can even lead to phototoxicity and thermal damage. Therefore, it is important to determine the suitable light source for the purpose and to describe the mechanism involved.

The first aim of the present study was to demonstrate the therapeutic effect of light therapy while proposing a safe light source without heat generation. We confirmed that 610 RLT using OLED induced activation of HSP90α and HSP27 in hMSCs through ROS control (Figure 2a,b). Furthermore, the +RLT group showed decreases of apoptotic factors and increases of angiogenic paracrine factors at both gene and protein levels compared to the nontreated group (−RLT). In cell viability analysis (Figure 2c–e), we found highly downregulated CASPASE‐3 and BAX/BCL2 expression in the +RLT group compared to the –RLT group. Since CASPASE‐3 represents the consequence of a series of apoptotic signaling events, downregulation of CASPASE‐3 could be interpreted as a result of negative regulation at one or more points within apoptotic cascades, including the pro‐apoptotic factor BAX/BCL2.^60^ In angiogenic capacity analysis (Figure 4), we found significant upregulation of VEGF, HGF, and FGF2 in the +RLT group compared to the –RLT group. We found that such increases of angiogenic growth factors secreted by the +RLT group stimulated the tubular network and migration efficacy of HUVECs, demonstrating an increased angiogenic efficacy of +RLT group. RLT‐treated hMSCs also showed better therapeutic outcomes when transplanted in vivo over no treatment (NT) and normal hMSCs injection (−RLT). After transplanting hMSCs to a mouse wound model, rapid recovery was observed for up to 3 days only in the +RLT group, which showed an early‐stage effect of cell therapy considering the survival rate of delivered cells (Figure 5c).^103^ Another intriguing tendency was found between 3 to 7 days, where wound ratio was drastically decreased in both +RLT and –RLT groups presumably due to increased angiogenic efficacy of stem cells as presented in Figure 4, which is critical in the proliferation step of wound healing.^104^ These results suggest that the higher cell viability of the +RLT group could allow them to survive longer at the wound site, thereby facilitating the sustained effect of angiogenic paracrine factors such as VEGF.

Overall, we showed that hMSCs therapy using RLT could enhance tissue regeneration from the perspective of cell viability and angiogenesis. While numerous studies have focused on how LLLT increases angiogenesis and viability, the mechanism by which LLLT regulates the activation of anti‐apoptotic and angiogenic factors in hMSCs mediated by HSPs has not been fully elucidated. Therefore, another aim of this study was to explore the mechanism underlying the anti‐apoptotic and angiogenic effects of RLT in association with HSP90α and HSP27. Based on experimentally identified factors, we here report the mechanism of RLT in respect of HSPs (Figure 6). Apoptosis‐inhibiting results can be explained by upregulated p‐Akt and NF‐κB cell survival signals confirmed in Figure 3.^72^, ^73^, ^83^, ^84^, ^85^ This p‐Akt‐NF‐κB signaling could also account for upregulated HIF‐1α, MMP‐2, and Cx43 in the +RLT group, which can synergistically drive upregulation of VEGF, HGF, and FGF2.^5^, ^74^, ^75^, ^86^, ^96^, ^97^, ^105^

## 4 CONCLUSION

We discussed the role and future clinical implications of light‐induced HSP90α and HSP27 with respect to skin wound healing. In light of this report, light therapy in combination with OLED could be a promising method to improve the wound‐healing efficacy of stem cell therapy by modulating intracellular ROS at mild levels in the wound model. Presenting the intracellular mechanism described in relation to OLED irradiation‐ROS‐HSPs, our results suggest that HSP90α and HSP27 are potential therapeutic targets in stem cell light therapy that can be used to manipulate survival and angiogenesis. Our RLT strategy might serve as a new paradigm for future clinical applications of cell therapy in the field of tissue regeneration.

## 5 MATERIALS AND METHODS

### 5.1 Cell culture

hMSCs were purchased from Lonza (Bazel, Switzerland) and cultured in low glucose Dulbecco's modified Eagle's medium (DMEM; Gibco BRL, Gaithersburg, MD, USA) supplemented with 10% (v/v) fetal bovine serum (Gibco BRL) and 1% (v/v) penicillin/streptomycin (Gibco BRL) at 37°C with 5% CO_2_. The cell culture medium was changed every 2 days. In this study, hMSCs within eight passages were used in the experiments. Human umbilical vein endothelial cells (HUVECs) were purchased from PromoCell (Heidelberg, Germany) and cultured in endothelial cell media (PromoCell) supplemented with Growth Medium SupplementMix (PromoCell) at 37°C with 5% CO_2_. The medium was changed every 2 days. Cells within four passages were used in the experiments.

### 5.2 OLED irradiation to hMSCs

An OLED panel was purchased from KANEKA Corporation (Tokyo, Japan). A power supply was installed in the cell culture incubator. The wavelength, voltage, current, and luminance of this light source were 610 nm, 3.5 V, 65 mA, and 115.40 Cd/m^2^, respectively. Energy density can be calculated based on voltage, current, and surface area of light source (8 cm × 8 cm, 300 J/cm^2^ for 24 h irradiation) from our previous study. The OLED was located under the culture plates, and the gap between the OLED and the culture plates was approximately 1 cm. Detailed and optimized conditions for applying OLED are described in Figure 1c. hMSCs were detached from the cell culture plate with trypsin (Gibco BRL) and resuspended in 500 μL media containing 10% fetal bovine serum. One day after, the culture medium was replaced with a fresh medium and cells were incubated for 24 h with OLED irradiation. Temperature of the culture medium was recorded after irradiation using an infrared thermal imaging system (FLIR i2, FLIR Systems Lne., Wilsonville, OR, USA). The medium extracted from each group was collected for analysis.

### 5.3 Intracellular ROS staining and analysis

To stain intracellular ROS, 2′,7‐dichlorodihydrofluorescein diacetate (DCF‐DA), (D339, Invitrogen, Carlsbad, CA, USA), a fluorescent indicator of ROS, was used. After red light treatment, cells were incubated with 10 μmol/L DCF in phosphate‐buffered saline (PBS, Gibco BRL) for 20 min at 37°C. After incubation, cells were washed twice with PBS and observed under a fluorescence microscope (DFC 3000 G, Leica, Wetzlar, Germany). The concentration of intracellular ROS was determined by measuring the fluorescence intensity (Ex/Em of 494/524 nm) using a microplate reader (Varioskan LUX multimode microplate reader, Thermo Fisher Scientific, Waltham, MA, USA).

### 5.4 TUNEL assay

After rinsing cells with PBS, a terminal deoxynucleotide transferase‐mediated deoxyuridine triphosphate nick end labeling (TUNEL) assay was performed with an ApopTag® Fluorescent In‐Situ Apoptosis Detection Kit (Millipore, Bedford, USA) to examine the apoptotic activity of hMSCs. After 4′,6‐diamidino‐2‐phenylindole (DAPI, Vector Laboratories, Burlingame, CA, USA) staining, TUNEL positive fluorescence was measured with a fluorescence microscope.

### 5.5 qRT‐PCR analysis

qRT‐PCR was performed to quantify relative gene expression levels of *human‐specific GAPDH, CASPASE‐3, BAX, BCL2, VEGF, HGF, FGF2, HSP90α, HSP 27, TNF‐α, NF‐κB, HIF‐1α, MMP‐2, mouse β‐actin, involucrin*, and *collagen type 1*. Total RNAs were extracted from samples using 1 mL TRIzol reagent (Life Technologies, Inc., Carlsbad, CA, USA) and 200 μL chloroform for each sample. Lysed samples were centrifuged at 12,000 rpm for 10 min at 4°C. Each RNA pellet was washed with 75% (v/v) ethanol in water and then dried. After drying, RNA samples were dissolved in RNase‐free water. For qRT‐PCR, a SsoAdvanceed™ Universal SYBR Green Supermix kit (Bio‐Rad, Hercules, CA, USA) and a CFX Connect™ real‐time PCR detection system (Bio‐Rad) were used. GAPDH (in vitro) and β‐actin (in vivo) served as internal controls.

### 5.6 Western blotting analysis

hMSCs were collected and lysed in radioimmunoprecipitation assay buffer (Rockland Immunochemicals Inc., Limerick, PA, USA). After centrifugation at 10,000*g* for 10 min, the supernatant was used as a protein extract. Protein concentrations were determined using a bicinchoninic acid assay (Pierce Biotechnology, Rockford, IL, USA). The same proteins from each sample were mixed with a sample buffer, loaded, and subjected to sodium dodecyl sulfate‐polyacrylamide gel electrophoresis (SDS‐PAGE; Hercules, CA, USA) using a 10% (v/v) resolving gel. Proteins separated by SDS‐PAGE were transferred to immuno‐blot polyvinylidene fluoride membranes (Bio‐Rad) and probed with antibodies against GAPDH (AF5718, 1:2000; R&D Systems, Minneapolis, MN, USA), CASPASE‐3 (#9662, 1:1000; Cell Signaling Technology, MA, USA), BAX (#5023, 1:1000; Cell Signaling Technology, MA, USA), BCL2 (ab182858, 1:2000; Abcam, Cambridge, UK), pAkt (#4060, 1:500; Cell Signaling Technology), Akt (#4691, 1:1000; Cell Signaling Technology), HIF‐1α (ab2185, 1:500; Abcam, Cambridge, UK), and Cx43 (#3512, 1:1000; Cell Signaling Technology) at 4°C overnight. Membranes were then incubated with horseradish peroxidase‐conjugated secondary antibodies (HAF008 for GAPDH; HAF008 for Akt, pAkt, BAX, BCL2, and Cx43; HAF017 for fibronectin) (all 1:1000 from R&D Systems) at room temperature for 1 h. Blots were then incubated in the dark. Luminescence was recorded on an x‐ray blue film (Agfa Healthcare NV, Mortsel, Belgium). Bands were imaged using an Image Studio Lite (LI‐COR Biosciences, OR, USA).

### 5.7 Enzyme‐linked immunosorbent assay

To analyze the secretion of angiogenic paracrine factors from hMSCs, we used ELISA kits for human VEGF, HGF, and FGF2 (R&D Systems) according to the manufacturer's instructions. CM was collected after 24 h of treatment with OLED. The optical density (OD) value of each well was measured at 450 nm using a microplate reader (correction at 540 nm; Tecan).

### 5.8 HUVEC tube formation assay

Tube formation of HUVECs was conducted using an Angiogenesis assay kit in vitro (ab204276; Abcam) following the manufacturer's protocols. The CM extracted from each group was mixed with HUVEC medium at a 1:1 ratio and then used to treat HUVECs cultured on pre‐coated Matrigel.

### 5.9 HUVEC migration assay

The CM extracted from each group was mixed with HUVEC medium at a 1:1 ratio and used to treat scratched HUVEC. A straight scratch was made on the HUVEC layer using a P1000 pipette tip (Neptune Scientific, San Diego, CA, USA). After incubation for 0 and 24 h, the gap width of the scratch re‐population was measured and compared to the initial gap size at 0 h. Using Adobe Photoshop CC (Adobe Systems, San Jose, CA, USA), the size of the denuded area was determined at each time point from each digital image. Bands were imaged using Photoshop CC.

### 5.10 Skin wound treatment assay

Eight‐week‐old female athymic mice (20–25 g body weight; Orient, Seoul, Republic of Korea) were used for the experimental groups to minimize variability in results caused by sex differences. Male mice are sometimes not preferred in wound‐healing research due to their tendency to remove the occlusive dressing (Tegaderm) resulting in wound bed drying.^106^ The mice were anesthetized using 200 μL xylazine (20 mg/kg) and ketamine (100 mg/kg) diluted in a normal saline solution. Two skin defect areas were induced with a 10 mm biopsy punch (LKF, Ansan, Republic of Korea) on the back of each mouse. The epidermis, dermis, and stratum corneum were then surgically removed. After skin removal, four 6–0 sutures (AILEE Co., Busan, Republic of Korea) were placed at the border of each wound to prevent wound collapse owing to skin contracture. Immediately after skin wound modeling, mice were subdivided into three groups (*n* = 6 per group): (1) no treatment [NT; wounds covered with the commercial skin dressing Tegaderm (3 M Healthcare, St. Paul, MN, USA)], (2) hMSCs [−RLT, no red‐light treatment (RLT); wounds injected with hMSCs + Tegaderm], and (3) RLT‐treated‐hMSCs [+RLT, with red light treatment, wounds injected with RLT‐treated‐hMSCs + Tegaderm]. In all groups except for the NT group, 4 × 10^5^ cells/100 μL were delivered per skin defect area. The untreated group served as a negative control. No medication or additional drug treatment was administered to the mice after surgery, and the wound‐healing process was observed for up to 10 days after treatment initiation. All animals were cared for according to the Guidelines for the Care and Use of Laboratory Animals of Sungkyunkwan University (SKKUIACUC2020‐01–12‐1, January 2020).

### 5.11 Histologic analysis

Impaired skin tissue specimens were retrieved 10 days after treatment. Whole wound tissue was fixed with 4% formaldehyde for 24 h and immersed with 30% sucrose for another 24 h. Skin tissue specimens were embedded in optimum cutting temperature (OCT) compound (SciGen Scientific, Gardenas, USA). Next, 12 μm sections were obtained from specimens and stained with H&E.

### 5.12 Immunohistochemical analysis

For immunohistochemical staining, samples embedded in OCT compound were cut into 10 μm‐thick sections at −22°C. To analyze the epidermis of the impaired skin tissue, sections were immuno‐stained with antibodies against involucrin (#924401, 1:1000; BioLegend) and counterstained with DAPI (Vector Laboratories, Burlingame, CA, USA). They were then examined using a fluorescence microscope (DFC 3000 G, Leica, Wetzlar, Germany).

### 5.13 Statistical analysis

All data are presented as mean ± SD. They were statistically analyzed using GraphPad Prism (GraphPad Software, San Diego, CA, USA). One‐way analysis of variance (ANOVA) and unpaired Student's *t*‐test were used to determine statistical significance. Differences with *p* < 0.01 or *p* < 0.05 were considered significantly different compared with the control.

## AUTHOR CONTRIBUTIONS

**Inwoo Seo:** Conceptualization (lead); data curation (equal); investigation (equal); methodology (equal); validation (equal); visualization (equal); writing – original draft (equal). **Sung‐Won Kim:** Data curation (equal); investigation (equal); writing – original draft (equal). **Jiyu Hyun:** Methodology (equal); validation (equal). **Yu‐Jin Kim:** Data curation (equal); visualization (equal). **Hyun Su Park:** Writing – review and editing (supporting). **Jeong‐Kee Yoon:** Funding acquisition (equal); writing – review and editing (supporting). **Suk Ho Bhang:** Funding acquisition (equal); supervision (equal); writing – review and editing (equal).

## CONFLICT OF INTEREST STATEMENT

The authors declare no conflicts of interest.

### PEER REVIEW

The peer review history for this article is available at https://www.webofscience.com/api/gateway/wos/peer-review/10.1002/btm2.10560.

## Supporting information


**Figure S1.** The original uncropped western blotting images corresponding to Figures 2e and 3b (caspase‐3, BAX, BCL‐2, p‐Akt, Akt, Hif‐1α, Cx43, and GAPDH). The strips marked with yellow boxes are the representative groups used in the article.

## Data Availability

Data available on request from the authors.
