# Supplementary material for: Enhancing viability and angiogenic efficacy of mesenchymal stem cells via HSP90α and HSP27 regulation based on ROS stimulation for wound healing
Source: Bioeng Transl Med. 2023 Jun 7;8(5):e10560. doi: 10.1002/btm2.10560 (PMC10487335; doi:10.1002/btm2.10560)
Supplement: Supplementary file 1 — Figure S1. The original uncropped western blotting images corresponding to Figures 2e and 3b (caspase‐3, BAX, BCL‐2, p‐Akt, Akt, Hif‐1α, Cx43, and GAPDH). The strips marked with yellow boxes are the representative groups used in the article. [file BTM2-8-e10560-s001.docx]

**Supporting Information**

**Enhancing viability and angiogenic efficacy of mesenchymal stem cells *via* HSP90*α* and HSP27 regulation based on ROS stimulation for wound healing**

*Inwoo Seo ^1^, Sung-Won Kim ^1^, Jiyu Hyun ^1^, Yu-Jin Kim ^1^, Hyun Su Park ^1^, Jeong-Kee Yoon ^2^, Suk Ho Bhang ^1,*^*

1School of Chemical Engineering, Sungkyunkwan University, Suwon 16419, Republic of Korea

2Department of Systems Biotechnology, Chung-Ang University, Anseong 4726, Republic of Korea

*Corresponding author:

Suk Ho Bhang, Ph.D., E-mail: sukhobhang@skku.edu, Tel.: +82-31-290-7242, Fax: +82-31-290-7272


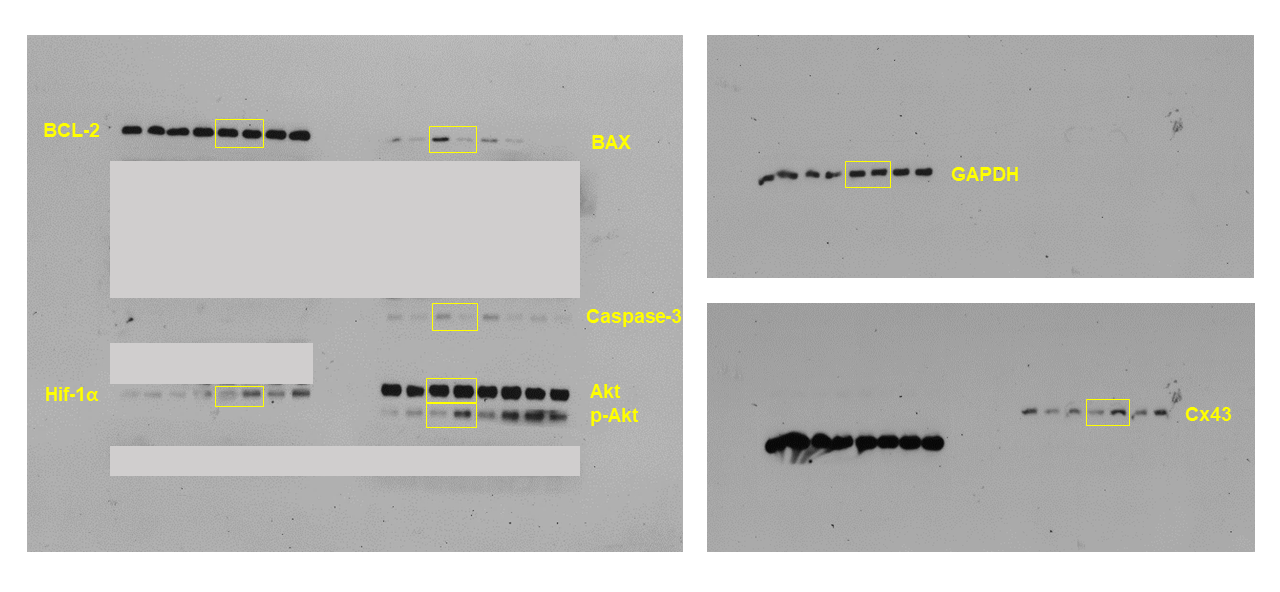


**Figure S1.** The original uncropped western blotting images corresponding to Figures 2e and 3b (Caspase-3, BAX, BCL-2, p-Akt, Akt, Hif-1α, Cx43, and GAPDH). The strips marked with yellow boxes are the representative groups used in the article.
